# Supplementary material for: High Expression of IRS-1, RUNX3 and SMAD4 Are Positive Prognostic Factors in Stage I–III Colon Cancer
Source: Cancers (Basel). 2023 Feb 24;15(5):1448. doi: 10.3390/cancers15051448 (PMC10000923; doi:10.3390/cancers15051448)
Supplement: Supplementary file 1 [file cancers-15-01448-s001.zip › cancers-2242485-supplementary.pdf]

cancers

MDPI

Supplementary Materials: High Expression of IRS-1, RUNX3 and SMAD4 are Positive Prognostic Factors in Stage I-III colon cancer

Hallgeir Selven [1,2], Lill-Tove Rasmussen Busund [3,4], Sigve Andersen [1,2], Mona Irene Pedersen [2], Ana Paola Giometti Lombardi [2] and Thomas Karsten Kilvaer [1,2,*]

1. Supplementary Material

Table S1. HC Procedure Discovery Ultra.

Baking	Deparaff- inization	Antigen retrieval	Primary Antibody	Secondary Multimer	Detection	Counterstain
Tissue 60°, 8min	Discovery wash 68°c, 3x12min	CC1 95°, 40min	IRS-1 1:800 36°, 32min	OmniMap anti-Rb HRP 37C°, 16min	ChromoMap DAB/ 37C°, 8/4min	Hem II/Blueing 37C°, 24/8min
Tissue 60°, 8min	Discovery wash 68°, 3x12min	CC1 95°, 40min	IRS-2 1:200 36°, 32min	OmniMap anti-Rb HRP 37C°, 16min	ChromoMap DAB/ 37C°, 8/4min	Hem II/Blueing 37C°, 24/8min
Tissue 60°, 8min	Discovery wash 68°, 3x12min	CC1 95°, 40min	SMAD4 1:200 36°, 32min	OmniMap anti-Rb HRP 37C°, 16min	ChromoMap DAB/ 37C°, 8/4min	Hem II/Blueing 37C°, 24/8min
Tissue 60°, 8min	Discovery wash 68°, 3x12min	CC1 95°, 40min	RUNX3 1:400 36°, 32min	OmniMap anti-Ms HRP 37C°, 16min	ChromoMap DAB/ 37C°, 8/4min	Hem II/Blueing 37C°, 24/8min

Abbreviations: CC1, cell conditioning 1; IRS, insulin receptor substrate; Rb, rabbit; DAB, Diaminobenzidine; RUNX,Runt-related transcription factor; Ms, mouse.

Table S2. Product information of antibodies and reagents.

Reagent	Reference	Company
IRS-1, EP263Y Rb monoclonal	Ab40077	abcam
IRS-2, EPR904(2) Rb monoclonal	Ab134101	abcam
SMAD4, EP618Y Rb monoclonal	Ab40759	abcam
RUNX3, 2B3 Ms monoclonal	Ab135248	abcam
Discovery Wash	7311079001	Roche
Ultra LCS (Liquid cover slip)	5424534001	Roche
Reaction buffer (Tris)	5353955001	Roche
Discovery CC1, Cell Conditioning (Tris)	6414575001	Roche
OmniMap anti-Ms HRP	5269652001	Roche
OmniMap anti-Rb HRP	5269679001	Roche
ChromoMap DAB kit	5266645001	Roche
Hematoxylin II	5277965001	Roche
Bluing reagent	5266769001	Roche
Ethanol 96%	20823.362	VWR, Avantor
Etnanol absolute	20821.296	VWR, Avantor
Xylene	28975.291	VWR, Avantor
Histokitt mounting medium	Assistant 1025/250	Sondheim/Rhoen, Germany

Abbreviations: Abbreviations: CC1, cell conditioning 1; IRS, insulin receptor substrate; Rb, rabbit; DAB, Diaminobenzidine; RUNX,Runt-related transcription factor; Ms, mouse.

Table S3. Dichotomized IRS-1, IRS-2, SMAD4 and RUNX3 in tumor and stromal cell nucleus and/or cytoplasm and their distribution over and correlation with clinicopathological variables (chi-square and Fisher's exact tests).

	T-C-IRS-1			T-C-IRS-2			T-N-SMAD4			T-C-SMAD4			T-N-RUNX3			T-C-RUNX3		
	L	*H*	*p*	*L*	*H*	*p*	*L*	*H*	*p*	*L*	*H*	*p*	*L*	*H*	*p*	*L*	*H*	*p*
Age			1.000			1.000			0.260			0.202			0.620			0.267
< 65	53	52		50	49		31	72		30	73		31	73		21	83	
> 65	159	159		152	153		75	239		70	244		84	231		47	268	
Gender			0.304			0.485			0.579			0.464			0.908			0.628
Female	108	119		104	112		55	173		51	177		61	165		39	187	
Male	104	92		98	90		51	138		49	140		54	139		29	164	
Weightloss			0.213			0.049			0.092			0.031			0.176			0.078
< 10%	106	122		99	119		50	174		47	177		58	168		29	197	
> 10%	49	40		50	35		29	61		30	60		30	58		19	69	
ECOG			0.147			0.989			0.285			0.094			0.968			0.833
0	107	115		109	107		50	166		43	173		59	159		35	183	
1	70	69		63	66		37	101		37	101		39	100		22	117	
2	28	22		25	25		15	37		15	37		15	35		9	41	
3	7	1		3	3		4	4		4	4		2	6		2	6	
Site			0.553			0.164			0.142			0.163			0.005			0.004
Sigm	106	108		89	110		45	166		41	170		47	164		23	188	
Transv	32	31		35	24		15	44		17	42		15	48		10	53	
Left	12	6		8	10		5	15		6	14		3	13		2	14	
Right	61	64		67	58		41	84		36	89		50	76		33	93	
pStage			0.073			0.129			0.141			0.024			0.132			0.456
I	25	42		25	39		11	55		8	58		23	43		11	55	
II	110	100		100	98		52	153		49	156		48	158		29	177	
III	77	69		77	65		43	103		43	103		44	103		28	119	
Grade			0.894			0.234			0.108			0.280			0.131			0.146
Well	16	16		17	15		12	20		10	22		13	18		4	27	
Mod	159	151		155	142		81	225		78	228		86	222		57	251	
Poor	32	38		25	40		12	57		12	57		15	54		6	63	
Und	2	2		2	2		0	4		0	4		0	4		1	3	
Vasc			1.000			0.880			1.000			0.762			0.520			1.000
No	109	75		88	85		49	132		47	134		49	133		33	149	
Yes	7	5		9	7		4	10		4	10		2	11		2	11	
Margins			0.551			0.472			0.771			0.456			0.290			0.541
0mm	14	10		9	17		7	18		4	21		8	17		2	23	
< 1mm	17	24		22	17		11	29		10	30		16	25		10	31	
1-2mm	15	18		15	15		10	22		10	22		9	22		4	27	
2-10mm	58	53		50	61		27	86		26	87		26	84		17	93	
10-50mm	81	66		72	64		41	102		41	102		34	110		21	123	
> 50mm	21	25		21	22		8	35		7	36		16	30		9	37	

	S-C-IRS-1			S-C-IRS-2			S-N-SMAD4			S-C-SMAD4			S-N-RUNX3			S-C-RUNX3		
	L	*H*	*p*	*L*	*H*	*p*	*L*	*H*	*p*	*L*	*H*	*p*	*L*	*H*	*p*	*L*	*H*	*p*
Age			1.000			0.183			0.842			0.805			0.810			0.276
< 65	22	83		56	43		53	50		36	67		65	39		31	73	
> 65	66	252		147	158		156	158		116	198		203	112		75	240	
Gender			0.862			0.545			0.256			0.742			0.548			0.706
Female	46	181		105	111		108	120		81	147		148	78		55	171	
Male	42	154		98	90		101	88		71	118		120	73		51	142	
Weightloss			0.158			0.013			0.556			0.151			0.684			0.151
< 10%	41	187		97	121		107	117		76	148		139	87		50	176	
> 10%	23	66		52	33		47	43		39	51		57	31		27	61	
ECOG			<0.001			0.472			0.418			0.121			0.524			0.141
0	33	189		110	106		102	114		69	147		140	78		48	170	
1	31	108		60	69		71	67		55	83		85	54		38	101	
2	20	30		29	21		27	25		22	30		33	17		16	34	
3	4	4		2	4		6	2		5	3		7	1		4	4	
Site			0.609			0.704			0.020			0.163			0.001			<0.001
Sigm	40	174		94	105		94	117		67	144		119	92		41	170	
Transv	15	48		31	28		27	32		21	38		38	25		10	53	
Left	3	15		9	9		9	11		7	13		13	3		4	12	
Right	30	95		67	58		77	48		55	70		97	29		51	75	
pStage			0.266			0.226			0.369			0.012			0.079			0.002
I	9	58		28	36		28	38		15	51		36	30		14	52	
II	46	164		96	102		104	101		73	132		129	77		40	166	
III	33	113		79	63		77	69		64	82		103	44		52	95	
Grade			0.199			0.127			0.003			0.088			0.019			0.116
Well	5	27		18	14		20	12		14	18		25	6		9	22	
Mod	71	239		155	142		161	145		117	189		202	106		83	225	
Poor	9	61		24	41		24	45		18	51		35	34		11	58	
Und	1	3		2	2		0	4		0	4		3	1		2	2	
Vasc			0.516			0.743			0.174			0.263			0.381			1.000
No	57	127		84	89		93	88		69	112		114	68		46	136	
Yes	2	10		9	7		10	4		8	6		10	3		3	10	
Margins			0.400			0.419			0.616			0.207			0.885			0.352
0mm	5	19		9	17		16	9		14	11		16	9		4	21	
< 1mm	7	34		22	17		23	17		18	22		28	13		13	28	
1-2mm	7	26		17	13		15	17		10	22		18	13		4	27	
2-10mm	18	93		51	60		54	59		36	77		67	43		29	81	
10-50mm	40	107		69	67		69	74		55	88		96	48		40	104	
> 50mm	10	36		24	19		23	20		14	29		30	16		14	32	

Abbreviations: T, tumor; C, cytoplasm; N, nucleus; S, stroma; L, low; H, high; ECOG, Eastern Cooperative Oncology Group.

Table S4. A) Univariate analyses of co-expression analyses between SMAD4 in tumor cytoplasm and RUNX3 in tumor nucleus (A1) and between SMAD4 in stromal cytoplasm and RUNX3 in and stromal cytoplasm (A2, log-rank test test, n = 452). B) Multivariable models including co-expressions of SMAD4 in tumor cytoplasm and RUNX3 in tumor nucleus (B1) and SMAD4 in stromal cytoplasm and RUNX3 in stromal cytoplasm (B2) and relevant clinicopathological variables (cox proportional hazards test, n = 452).

A)	A1 Tumor N(%)	5 Year	Median	HR(95%CI)	P	A2 Stroma N(%)	5 Year	Median	HR(95%CI)	P
SMAD4/RUNX3					< 0.001					< 0.001
SMAD4-/RUNX3-	30(7)	57	70	1		70(15)	63	182	1	
SMAD4+/RUNX3-	35(8)	72	NA	0.56(0.2-1.58)		32(7)	57	NA	1.09(0.42-2.83)	
SMAD4-/RUNX3+	65(14)	73	NA	0.58(0.22-1.49)		76(17)	79	NA	0.47(0.24-0.94)	
SMAD4+/RUNX3+	272(60)	84	NA	0.33(0.14-0.75)		224(50)	88	NA	0.29(0.16-0.51)	
Missing	50(11)					50(11)				

B)	B1		B2	
SMAD4/RUNX3				
SMAD4-/RUNX3-	1		1	
SMAD4+/RUNX3-	0.54(0.23-1.27)	0.157	1.36(0.69-2.68)	0.372
SMAD4-/RUNX3+	0.65(0.31-1.35)	0.243	0.5(0.27-0.93)	0.030
SMAD4+/RUNX3+	0.34(0.18-0.66)	0.001	0.34(0.2-0.57)	< 0.001
Age	1.03(1.01-1.05)	0.004	1.03(1.01-1.05)	0.007
pTNM				
I	1		1	
II	1.91(0.73-5)	0.187	2.09(0.8-5.46)	0.133
III	5.28(2.07-13.44)	< 0.001	4.97(1.96-12.62)	<0.001
Margins				
0mm	1		1	
< 1mm	0.5(0.22-1.14)	0.098	0.56(0.25-1.26)	0.160
1-2mm	0.14(0.04-0.51)	0.003	0.18(0.05-0.64)	0.008
2-10mm	0.35(0.17-0.72)	0.005	0.37(0.18-0.79)	0.010
10-50mm	0.46(0.23-0.91)	0.025	0.54(0.27-1.07)	0.078
> 50mm	0.34(0.13-0.88)	0.026	0.35(0.13-0.9)	0.029

Abbreviations: Abbreviations: CC1, cell conditioning 1; IRS, insulin receptor substrate; Rb, rabbit; DAB, Diaminobenzidine; RUNX,Runt-related transcription factor; Ms, mouse.

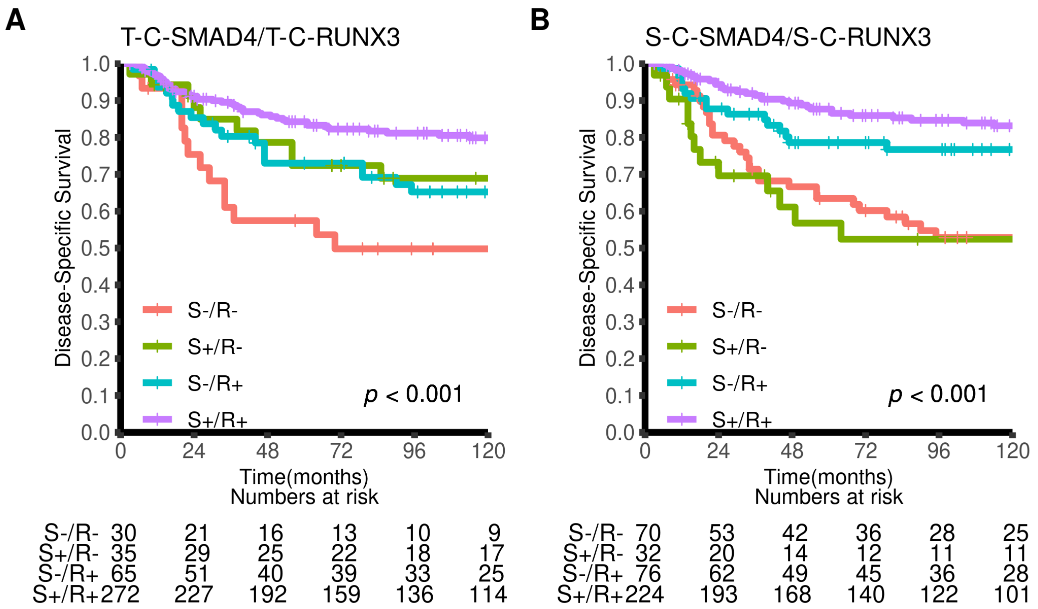

Figure S1. Disease-specific survival curves for the co-expressions between A) SMAD4 in tumor cytoplasm and RUNX3 in tumor nucleus and B) SMAD4 in stromal cytoplasm and RUNX3 in stromal cytoplasm.
